# Pre-graduation grade inflation in medical training in Turkey: a longitudinal study from 2005 to 2020

**DOI:** 10.1186/s12909-021-02819-0

**Published:** 2021-07-22

**Authors:** Engin Karadag

**Affiliations:** Akdeniz University, Campus, 07070 Antalya, Turkey

**Keywords:** Medical training, Higher education, Pre-graduation medical training, Grade inflation

## Abstract

**Background:**

Grade inflation which is known as the awarding of higher grades than students deserve in higher education has been observed since the 1960s. There is comprehensive evidence that document the allegations, prevalence, and severity of grade inflation in higher education in universities around the world for the past 10 years.

**Methods:**

This study analyzes the change in the ratio of graduates with a “very good (>2.99)” degree in medical education in Turkey within a 15-year-long period in terms of the grade inflation (when all other factors are constant), and factors that affect the overall achievement grades. The analyses were carried out using the grade point average (GPA) of 9,618 students who graduated from the medical schools of 25 Turkish universities, and grades of 288,540 students for 7,597 courses. In doing so, the “real” university random effects estimator modelling considering the differences in universities with correlation, ANOVA, t-test and ANCOVA analyses were carried out.

**Results:**

The results revealed that there was a marginal increase in grades in medical training before graduation. Twenty-nine percent grade inflation was detected in line with the relevant findings in literature and this figure is one of the highest that has been reported so far. It was also detected that the ratio of graduates with a “very good (>2.99)” degree was 17% in 2005 and it increased to 46% in 2020. Additionally, the class size, academic rank of the instructors, grades, course contents, types of the universities (public & non-profit private), accreditation of the program, and the age of the medical schools were considered as important determinants of the difference in course grades.

**Conclusion:**

These results show that both the uncontrolled expansion of medical schools in Turkey and the decrease in quality cause a significant increase in grades. Moreover, an important finding is that accreditation slows down the grade inflation. Both the course grades following the accreditation process and the inflation in the graduation grades (grade inflation) slowed down significantly in the accredited medical schools. This finding is an important example for the necessity of accreditation for universities, which is referred to as the “gold standard” to improve the quality of medical education.

## Introduction

Pre-graduation medical training assessment is a critical part of testing the acquired academic competencies and skills. However, the most important component that is mostly disregarded in the assessment process is the validity of course grades. There is a general opinion in the literature that grading in medical education is standardized insufficiently and is often subjective [1]. In this regard, grade inflation is a process that reduces the real value of grade A to a medium grade [2]. The grade inflation weakens the common standards; thus, it becomes difficult to compare grades with students’ knowledge and competencies [3]. Inflating the grades increases the tendency to wonder what the essence of the grade is [4]. Lately, there has been a growing awareness that grade inflation might cause a greater problem compared to previous years. These thoughts result in a common subject: How will the sufficiency of the assessment be guaranteed to measure students’ progress? Moreover, if students get higher scores than previous years on average, could this stem from the outcome of better homework, a more planned syllabus or communicating more with other students about the requirements of the course?

Although grade inflation is not a problem concerning only a single academic discipline, the number of studies that answer these questions and are about grade inflation is limited in the relevant literature compared to other issues. Therefore, the focus of this study is to examine the change in the ratio of graduates with a “very good (>2.99)” degree in Turkish medical education, “grade inflation” and factors that affect course grades considering the need for further studies on whether this phenomenon exists, and if it does, what consequences of this inflation in medical education circle are observed? The data for this study included the Grade Point Average (GPA) of 9,618 graduates of 25 universities for 15 years-between 2005 and 2020, and 288,540 student grades for 7597 courses. The number of studies on “grade inflation in medical education” is quite limited; thus, the methodology and the findings of the current study were planned to help the re-consideration of the concerns about “grade inflation”, and whether they are critical or not.

## Background

### Medical training in Turkey

The origins of pre-graduation medical education in Turkey stemmed from Istanbul University School of Medicine established in 1933. Following Istanbul University; Ankara University, Ege University, Atatürk University, Hacettepe University, Dokuz Eylül University, and the medical schools of these universities were founded, and medical training was started in each school by adopting a similar education model. As of 2021 (February), 95 thousand students have received pre-graduation medical education in 104 medical schools, 77 of which belong to state universities and 27 of them belong to non-profit private universities. 35,700 instructors, 16,500 of whom are academics, work in these medical schools in total.

The universities in Turkey are divided into two as public and non-profit private universities. University education is free of charge in all programs of public universities (including the pre-graduation medical training) in Turkey. Education programs of non-profit private universities require a considerable amount of tuition fee. The yearly tuition fee of the medical schools in non-profit private universities changes between 58,000 TL and 151,000 TL (*M = 93.80*, *SD* = 23,93). However, it is mandatory for non-profit private universities to spare at least 15% of the total student capacity to beneficiary students for each program.

Students’ placement to medical schools in Turkey is carried out via the results of a central examination conducted by Assessment, Selection and Placement Center (ÖSYM). This high-stakes exam, which consists of multiple-choice test items, covers the fields of Turkish language, mathematics, science, and social sciences. Students who get the highest scores in the exam, which is taken by 2.5 million candidates annually, are placed in medical schools. For example, in 2020, the total quota of medical schools was 16 thousand, and among 2.5 million candidates, the rank of the students placed in state university medical schools was 19 thousand, and the rank of the students placed in non-profit private university medical schools was 47 thousand. These results indicate that the most distinguished and successful students preferred the medical schools in Turkey. In addition, this situation is not a new phenomenon and has been observed in medical schools since their establishment.

In pre-graduation medical education in Turkey, the Anglo-Saxon model has been adopted. In this model, after 5 years of theoretical, applied, and clinical training, students receive only clinical practice (internship) in the sixth year (final year). However, due to the foreign language preparatory program of some medical schools where the medium of instruction is English, the study period can be extended up to 7 years. For a medical school to start educational and instructional processes in Turkey, there are some prerequisites which require a pre-determined physical infrastructure accordance with the number of students, fully equipped-educational materials- laboratories (Medical Biology, Medical Genetics, Anatomy, Histology and Embryology, Physiology, Medical Biochemistry, Medical Microbiology, Pharmacology, Biophysics) and Medical (Vocational) Skills Laboratories, classrooms, administrative structures, library, conference hall -all of them completed- and 19 instructors, of whom at least 12 have got tenure [5]. Currently, in Turkey, pre-graduation medical education is conducted with different educational models (conventional, integrated, interactive, hybrid) [6].

In the classical education model, subjects are handled individually and separately without being linked to other courses. This model is still used in a small number of medical schools, due to its extensive content and not linking basic sciences with clinical sciences and professional contexts. The integrated model was developed in the 1950s in the US and was launched under the leadership of Hacettepe University School of Medicine in Turkey in 1960s. This system connects the knowledge of different disciplines related to each other and facilitates integration. Additionally, problem-based learning in medical education was initially developed at McMaster University in Canada in 1967. In Turkey, it was applied for the first time between 1997 and 1998 at Dokuz Eylül University School of Medicine. In this model, beyond the theoretical knowledge, the human body is taken into consideration from all aspects over a pre-determined disease scenario [7].

Assessment and evaluation practices in the pre-graduation medical schools in Turkey are generally conducted in a way including multiple components in pre-clinic and clinic periods, although there are some differences from time to time. In these assessment and evaluation processes, multi-choice tests, clinical oriented reasoning examination (CORE), objective structured clinical examination (OSCE), objectively structured clinic exams, and educator evaluation forms are used. During the internship period, students participate in the clinical practice and academic studies of their departments, and the art of medicine is matured by diagnosis, treatment, and patient follow-up. Practice exam, theoretical exam, OSCE, structured oral examination, classical oral examination and chairside practice exams are utilized for the assessment and evaluation in this period.

National Medical Education Accreditation Board (NMEAB) established by the Council of Deans of Medicine in 2008 in Turkey set the National Standards of Pre-graduation Medical Education and has launched the accreditation of medical training programs in 2010. In 2011, on the demand of Council of Higher Education (CHE) that NMEAB must be an independent organization, Medical Education Programs Evaluation and Accreditation Association (MEPEAA) was established registered by CHE [8]. The association is a quality agency recognized by the Higher Education Quality Committee and World Federation for Medical Education and carries out national and international medical education accreditation operations. The accreditation process of medical schools in Turkey started in 2011, and the accredited education programs are carried out in 41 of the existing 104 medical schools. Considering the “Pre-graduation Medical Education Standards” renewed in 2021, under nine main headings and 23 sub-headings, there are 66 fundamental standards that must be met and 29 development standards that are recommended to be met. Main topics are: (*i*) aims and objectives, (*ii*) the structure and content of the education program, (*iii*) evaluation of students, (*iv*) students, (*v*) program evaluation, (*vi*) academic staff, (*vii*) infrastructure and facilities, (*viii*) organization, management, and execution, (*ix*) continuous innovation and development (see 8).

### Grade inflation

Students receive scores to indicate their academic success [9, 10]. When the grades are inflated, the tendency to question the rationale behind the grading increases [4, 11]. In this respect, “*grade inflation*” is a common explanation for rising grades in terms of giving higher grades to an equivalent work. Additionally, empirical literature on grade inflation in higher education outside the US is inadequate, this case has been widely investigated in the US [12, 13]. Over the last few decades, *grade inflation* claims in higher education have increased in US universities, along with extensive evidence documenting its prevalence and seriousness. It is known that students spend less time studying in grade-inflated classes. Additionally, students who receive inflated grades in entry-level or prerequisite classes often state that they feel inadequate in advanced courses and present the effects of grade inflation as the main reason [14].

Many factors influence the increase in grades in higher education. For example, Schutz et al. [15] attributed this increase to three factors: (a) student evaluation of classes became mandatory, (b) students became increasingly career-oriented, and (c) learning outstripped family income. Some researchers reported that the need to improve the registration of certain undergraduate programs [16–21] also triggered the elevation of grades. William, Li, and Wing [22], Tampieri [23], and De Witte, Geys, and Solondz [24] added that competition between colleges also encourages grade inflation to put students in better jobs. Moreover, the additional resources provided to state institutions may also lead to grade inflation. Hernández-Julián [25] showed that grade-dependent scholarships can lead students to search for easier classes to maintain the required grade point average.

To sum up, grade inflation refers to a tendency to reduce academic requirements and give students higher grades than they deserve [26]. Additionally, grade inflation is a process followed by higher education institutions that reduces the actual value of A grade to an average grade value [2]. Grade inflation weakens standards, making it difficult to compare grades with knowledge and qualifications [3]. From this perspective, Crumbley, Flinn, and Reichelt [4], described grade inflation in higher education institutions as a “fatal symbiosis”. Unfortunately, there is no consensus and reliable proof on the causes and consequences of grade inflation. Regardless of the reason, there are two issues related to the phenomenon of grade inflation in contemporary higher education. The first is related to the fact that the distribution of letter grades increases (inflation) over time, and thus, A and B are given more than C, D, or F. The second is the potential factors affecting the course grade.

### Hypotheses

The aim of this study is to examine the reasons of the change in the ratio of graduates with a “very good (>2.99)” degree in pre-graduation medical training in Turkey and the potential factors that affect the course grade. This main objective composes the first hypothesis of the study which can be formulated as **H**_**1**_. The ratio of graduates with a “very good (> 2.99)” degree from the pre-graduation medical training in Turkey has been increasing. The second hypothesis is: There is grade inflation in the pre-graduation medical training in Turkey (**H**_**2**_). Students’ gender, the type of the universities (public or non-profit private), accreditation of the program, age of the medical schools, medium of instruction, and differences in university entrance scores, which were assumed to affect grades, were disregarded to validate this hypothesis. Thus, absolute grade inflation was calculated. The last hypothesis is the **H**_**3**_: the class size, the academic rank of the instructors, grade, content of the course, types of the universities (public & non-profit private), accreditation of the program, and the age of the medical schools have an impact on course grades. Therefore, research questions of the study seek to investigate the following questions:

1. What is the ratio of those who graduated with a “very good (>2.99)” degree from the pre-graduation medical training in Turkey?
2. Is there grade inflation in the pre-graduation medical training in Turkey?
3. Do class size, the academic rank of the instructors, grade level, content of the course, types of the universities (public & non-profit private), accreditation of the program, and the age of the medical schools have an impact on the course?

## Methodology

The data of this study included the students of 25 universities’ medical schools in Turkey. The criterion for determining those universities is the university entrance percentile rankings. The institutions were ranked based on their percentiles and divided into five groups considering the percentiles of the last student admitted to their medical schools (The phrase “the last student admitted to the program” refers to the central university entrance exam and the admission process. All the students receive their university entrance exam scores and a relating percentile. The students are admitted to the programs based on their central exam scores. The universities were categorized according to the student with the lowest score they admitted. For example, a medical school which accepts a student with the lowest score of 534.81 (out of 575) means that the scores of the rest of the students are above this threshold.). Lastly, the study was carried out using the data (grades) obtained from 25 medical schools, five of which are added each entry-level. The data included the years between 2005 and 2020. The data included a 15-year long period between the 2004–2005 academic year and 2019–2020 academic year. Two types of data were used in the study. Firstly, the GPAs of students who graduated within the abovementioned 15-year-long period were used to examine the grade inflation and the change in the ratio of graduates with a “very good (>2.99)” degree. This data set included 9,618 students’ grades. With Turkey’s signing up for the Bologna Process, graduation grade in universities was changed to the 4-point grading scale. The graduation grades before 2002, which were based on 100-point grading scale, were converted into 4-point grading scale in accordance with the values presented in Table 1. Secondly, the grade data that were formed at the end of the academic year for each course were used to determine the factors that affect course grades. This data set included 288,540 students’ grades for 7,597 courses. The semantic (between AA-FF) letter system of the medical schools was converted to calculate the mean grade in each course, so the mean grade may change between 4.0 (a course where all students got AA) and 0 (a course where all students got FF) (see Table 1). In the student information systems, there is no information about how the exams are carried out (open-ended, multiple choice, etc.) However, very similar tests are used in the context of courses in pre-graduation medical education in Turkey. In addition, it could be stated that this obscurity is one of the most important limitations of the present study.

**Table 1:** *Grade categorization system*

100-point grading scale	4-point grading scale	Letter Grade
88–100	4.00	AA
81–87	3.50	BA
74–80	3.00	BB
67–73	2.50	CB
60–66	2.00	CC
53–59	1.50	DC
46–52	1.00	DD
35–45	0.50	FD
0–34	0.00	FF

Firstly, the gender of the graduates, the percentile of the last student admitted to the program, some university properties (the type of the university (public or non-profit private), program accreditation, age of the medical schools, medium of instruction) were included in the analyses regarding the grade inflation and the changes in the ratio of graduates with a “very good (>2.99)” grade in this study. While the empirical literature on grade inflation generally uses production functions in education, it does not control the impact of changes in university and student differences on the improvement of student grades. Therefore, the “grade inflation” observed in most of these studies may be the result of the fact that universities become more technically efficient in teaching and learning, and students in learning.

The “real” (university and student) random effects estimator (REE) developed by Greene [27] was used to consider the differences in universities and students in terms of the year. This tool develops older stochastic random effects panel models to separate the changes of some properties of the university in time from the heterogeneity of the university profile [28]. In the analyses, the variables of the gender of the graduates and the percentile of the last student admitted to the program and the university characteristics (type of universities (state & non-profit private), accreditation of the program, age of the medical schools, medium of instruction) were added as dummy variables. For each dummy variable, k (category) -1 dummy variables were generated to avoid multicollinearity. For dummy variables, the reference value to the value excluded and the fit values of the remaining groups represent the difference from this reference.

The literature presented in the previous section reveals that grade inflation differs according to class population, academic history of the instructor, grade level, fields, and university entry scores. These findings support the fact that many independent factors could affect the average grade given in a particular course. In this study, eight potential bias factors were examined: classroom population, academic history of the instructor, grade level, content (field), types of the universities (public & non-profit private), the accreditation of medical schools and medical schools’ age.

Each factor was examined one by one using correlation, ANOVA, and t-tests, and the factors associated with the average course grade are included in the final analysis (ANCOVA) as a common variable. Including these factors in the analysis as common variables, when testing the main relationship, meaning comparing the grades given before the setting, provides data to check the side effects that the main relationship may cause.

## Results

### Graduates with the “Very Good (>2.99)” degree

**Fig. 1:**
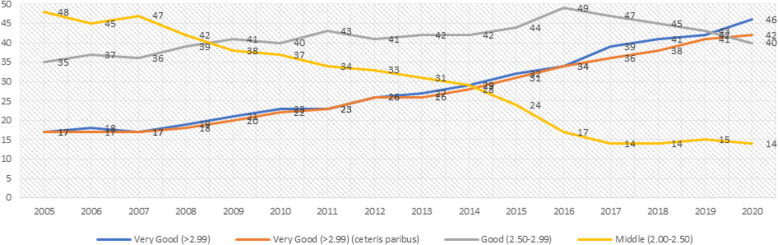
The graduation grade classification of all students

It was found that the ratio of graduates with the “very good (>2.99)” degree in Turkey was 17% in 2005 and it increased to 46% in 2020. It was also found that the ratio of graduates with a “good (2.50–2.99)” degree increased from 35 to 40% in this period whereas the ratio of graduates with a “moderate (2.00–2.49)” degree decreased from 48 to 14% (Fig. 1).

The change in the ratio of those who graduated with a “very good” degree was examined using the stochastic limit coefficient estimations of REE (Table 2). The results showed that the coefficient, which indicated that female students are more likely to graduate with a “very good” degree compared to male students, is statistically significant. This result indicates that being a woman has a positive and significant effect on performance. Point estimation shows a 1% increase in the ratio of women among the students increased the average of those who graduated with a “very good” degree at the ratio of 0.09% (when all other factors are constant). Moreover, a significant and positive correlation was found between the percentiles of the last student admitted to the medical schools (high entrance score increases as the percentile decreases) and the ratio of those who graduated with a “very good” degree. For example, a 10% increase in the entrance percentiles (entrance with a lower score) increases the ratio of those who graduated with a “very good” degree at the ratio of 2.5% (when all other factors are constant). This also showed that a university that accepts students from the high percentile (with a low score) will graduate 4% more students with a “very good” degree compared to a university that accepts students from the low percentile (with a high score).

**Table 2:** Standard random effects estimates

Variable name	Random Effects
*Students’ characteristics*	
ln (*%* Female)	0.09 (0.079)*
ln (percentile of the last student admitted)	−0.25 (0.041)*
*University characteristics*	
Non-profit private universities	0.57 (0.189)*
Accredited university	−0.27 (0.125)*
English medium instruction programs	0.11 (0.098)*
Medical School Age	−0.17 (0.081)*
σi	0.071
σe	0.063
rhoi	0.619
Within-R^2^	0.432
Observations	9.618
Number of universities	25

*p < 0.001

Robust standard errors corrected for clustering by university are reported in parentheses. σi and σe are the estimated standard deviations for the fixed effects and the error term, respectively, ρi is the fraction of the variation in the dependent variable accounted for by the fixed effects and ρ is the correlation between the fixed effects and the included variables

The results obtained in the context of university properties can be summarized as follows. The coefficient, which indicates that it is more likely for students in non-profit private universities to graduate with a “very good” degree than students in public universities, is statistically significant. The point estimation shows that a 1% increase for the students in non-profit private universities increased the average of those who graduated with a “very good” degree at the ratio of 0.57% (when all other factors are constant). The coefficient, which indicates that it is more likely for students of a nonaccredited university to graduate with a “very good” degree than students in an accredited university, is statistically significant. The point estimation shows that a 1% increase for the students of a nonaccredited university increased the average of those who graduated with a “very good” degree at the ratio of 0.27% (when all other factors are constant). The coefficient, which indicates that it is more likely for students in English medium instruction programs to graduate with a “very good” degree than students in Turkish medium instruction programs, is statistically significant. The point estimation shows that a 1% increase for the students in English medium instruction programs increased the average of those who graduated with a “very good” degree at the ratio of 0.11% (when all other factors are constant). A significant and negative correlation was found between the ages of medical schools and the ratio of those who graduated with a good degree. For example, the point estimation shows that a 10% increase in the age of the medical schools decreases the average of those who graduated with a “very good” degree at the ratio of 0.17% (when all other factors are constant). This also showed that young schools will graduate 6% more students with a “very good” degree compared to relatively older schools.

Next, a continuous increase was observed in the estimated coefficients in year dummies. Additionally, estimated coefficients for year dummies are statistically significant as of 2009 after checking the features of students and universities (*p < .*001). The point estimation (when all other factors are constant) showed that the ratio of those who graduated with a “very good” degree, which was 17% in the 2005 academic year, increased to 45% with a 25% increase (Fig. 1); and this means that it explains almost all of the 29% increase in the ratio of those who graduated with a “very good” degree before checking the variables. These results indicate that there has been a significant grade inflation in the pre-graduation medical training in Turkey within the last 15 years.

### Grade inflation

**Fig. 2:**
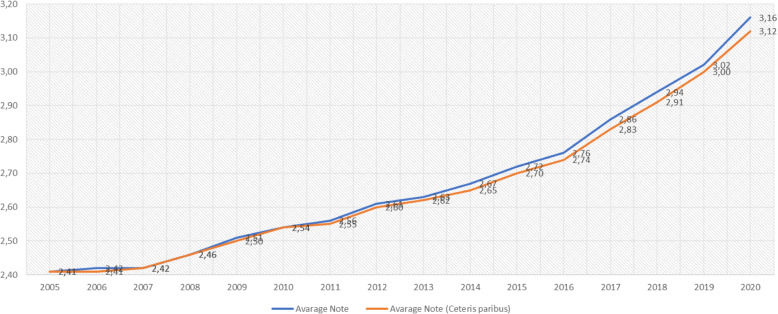
The graduation grades of all students

While the students in the medical schools in Turkey graduated with an average GPA of 2.41 (*SD* = 0.29) in 2005, this average increased to 3.16 (*SD* = 0.58) in the past 15 years (2020). This detected difference is quite high and statistically significant (*t* = 21.37; *p < .*001). Accordingly, a marginal increase of 31.12% in the graduation grades between 2005 and 2020 was detected, and this finding is an indicator of high-grade inflation in the pre-graduation medical training in Turkey. Considering the grade averages, there was an increase in each year compared to the previous year (except 2007). The highest-grade inflation on yearly basis was in the grades of students who graduated in 2020 with 4.64%, that is followed by students who graduated in 2017 with 3.62% (Fig. 2).

The results of the former analysis showed that the following six factors were associated with the graduation grade: gender, the percentile of the last student admitted to the program, university types (public or non-profit private), program accreditation, age of the medical schools and medium of instruction. The average values corrected according to these six factors were calculated with the ANCOVA for each year (Fig. 2). A marginal increase of 29% was detected in the graduation grades in terms of the corrected averages, but there is a high-grade inflation in the pre-graduation medical training in the past 15 years even when the factors that affect the grades are taken under control.

### Factors that affect course grade

#### Class size differences

The total number of students in the class is the top factor that affects grades. Thus, the correlation coefficient of the relationship between class sizes and average grades was examined. The results showed a significant and negative correlation between average grade and class size (*r* = −.36, *p <* .001). In other words, it can be concluded that the fewer the students in the class, the higher their average grades are.

#### Differences regarding the academic rank of the instructor

Another potential factor that affects the course grade is the academic rank of the instructors. All the lessons in medical education in Turkey are offered by instructors with a doctoral degree (Ph.D. & M.D.) and there is a hierarchical ranking among instructors as assistant professor, associate professor, and professor. In the data set of the study, 71% of the lessons were given by instructors with the highest two titles (associate professor and professor). According to the results of ANOVA, the average grade (*M =* 2.93*, SD* = 0.98) in lessons offered by instructors titled professors was higher than the average grades in lessons offered by associate professors (*M =* 2.87, *SD* = 0.95) and assistant professors (*M =* 2.61, *SD* = 0.91) (*F* = 47.29; *p* < .001). In conclusion, the academic titles of the instructors affected the course grades significantly.

#### Grade differences

Another potential deviation factor is the grade level. The number of students is quite high in the first or second-grade courses because of the students who failed the class and the upper-grade students who were unable to continue their classes. According to ANOVA results, there is a significant difference between the grades and average course grades (*F =* 59.78, *p <* .001). The senior year (6th Grade) classes have the highest average grade (*M =* 3.47, *SD* = 0.54) followed by the fifth (*M =* 3.21, *SD* = 0.66) and fourth (*M =* 3.01, *SD* = 0.69) grades. The first grade (*M =* 2.13, *SD* = 0.89) has the lowest average grades followed by the second (*M =* 2.41, *SD* = 0.88) and third (*M =* 2.74, *SD* = 0.91) grades. In conclusion, the grade level affects the course grades as well.

#### Content (field) differences

It is known that both the competencies expected from the students and the lessons they take are more complex and difficult according to the requirements of the courses. For example, the courses in fields like surgical medicine, where courses like medical pathology are predominant, are relatively more difficult than courses in basic medical sciences and require students to spend more effort. In this regard, another factor that potentially affects the grades is the field of the course. The differentiation of average grades of the courses based on their fields (Basic Medicine, Internal Medicine and Surgical Medicine) was examined with the ANOVA. The results showed that there was a difference between average course grades in terms of the field of the course (*F =* 39.27, *p < .*001). The lowest grades were given in basic medicine (*M =* 2.31, *SD* = 0.93) while the highest grades were given in surgical medicine (*M =* 3.07, *SD* = 0.59). The average grade in internal medicine was 2.87 (*SD* = 0.63). Accordingly, it is observed that the field of the course affects the course grade.

#### University differences

Another factor that potentially affects the grades is the type of university (public or non-profit private). In this regard, the differentiation of average course grades of the programs based on the type of the university was examined with the t-test. The results showed that there was a difference between the grades given in public universities and grades given in non-profit private universities (*t =* 29.33, *p < .*001). The course grades in non-profit private universities (*M =* 3.11, *SD* = 0.86) are quite higher than the course grades in public universities (*M =* 2.54, *SD* = 0.82). In conclusion, the type of university affects the course grade.

#### Accreditation differences

Accreditation expresses the assessment and external quality assurance process that measures whether the pre-determined academic and field-specific standards are met by a higher education program. In this regard, it is an expected process for accredited higher education institutions to have a higher standard in both educational and assessment-evaluation processes. Thus, another factor that potentially affects the grades is the accreditation of the program. In this regard, the differentiation of average course grades of the programs based on whether the university is accredited was examined with t-test. The results showed that there was a difference between the grades given in accredited programs and the grades given in non-accredited programs (*t =* 21.93, *p < .*001). The course grades in non-accredited programs (*M =* 3.11, *SD* = 0.86) are significantly higher than the course grades in accredited programs (*M =* 2.76, *SD* = 0.79). In conclusion, it could be summarized that the accreditation of the program affects the course grades.

#### Age differences of the medical school

The age of the medical schools is closely related to various variables from the recruitment of lecturers and physical opportunities to the quality of various educational programs. The ages of the schools are among the top factors that affect grades. The sufficiency of lecturers and schools are more limited in relatively younger schools; thus, expectations from students tend to be lower in such schools. For this reason, the correlation coefficient of the relationship between the ages of schools and average course grades was examined. The results showed a significant and negative correlation between the age of the medical schools and average grades (*r* = −.62, *p <* .001). Accordingly, the younger the schools are, the higher the average course/lesson grade is.

## Discussion and conclusion

The aim of the present study was to examine the grade inflation in higher education literature, which has been neglected so far. Moreover, the study examined the change in the ratio of graduates with a “very good (>2.99)” degree within the past 15 years, grade inflation (when all other factors are constant) and the factors that affect course grade. Therefore, the study could be considered as an authentic study compared to the other related studies in the literature and provides important proofs about the pre-graduation medical training in Turkey.

Many countries use accreditation as a regulatory mechanism to improve the quality of medical education [29]. One of the most important results obtained is about the effect of accreditation on the grade inflation. Both the course grades following the accreditation process and the inflation in the graduation grades (grade inflation) slowed down significantly in the accredited schools. In this regard, it could be an important example of the necessity of accreditation, which is referred to as the “gold standard” to improve the quality of medical education [30]. Additionally, it can be stated that accreditation reflects the guarantee that the program manages the education and learning effectively leaving aside the accreditation debate in higher education. Therefore, medical school’s accreditation can serve as a tool to increase medical specialty and to encourage communication and interaction with society [31].

Various studies have shown that student grades are related to the academic rank of the instructor. Research on this matter has demonstrated that instructors with a lower rank consistently give far higher marks than instructors with a higher rank [32–35]. But the findings obtained in the study are not compatible with the relevant literature. It was found that the highest grades were given by the professors and the associate professors and the lowest grades were given by the assistant professors in Turkey. There are several possible explanations for this controversy. Introductory courses in Turkey are not preferred to be given by professors and associate professors compared to Anglo-Saxon countries, and these courses are mainly conducted by assistant professors. The findings of this study and results in the literature demonstrate that the lowest grades are given for the introductory courses. Because the number of students is relatively higher due to retakes and the difficulties that junior students face during the adaptation period to the educational program. Another possible explanation comes from the cultural background of Turkish higher education. In Turkey, especially professors in faculties, do not devote much time to the assessments [36]. The assessments are often carried out by their assistants. Another finding that should be interpreted along with this result is that the lowest marks are given to courses offered in the first year. It is also related to Turkish culture, to give low grades compared to other classes in first-grade courses. Instructors tend to punish students implicitly for potential problems that may occur in the future, based on the proverb “you should crush the head of the snake when it is young”, that is why the reasons are still cultural.

The results of this study reveal that the ratio of those who graduated with a “very good (>2.99)” degree in the medical schools in Turkey in 2020 increased compared to 2005. This result is compatible with the literature. According to a recent study conducted by Hernández-Juliána and Looneyb [37], 24% of the grades were A, 35% were B, 27% were C, 9% were D and 4% were F in 1982. The ratio of A increased to 38% in 2001 while the ratio of D decreased to 6%. The study also revealed that the factor that affects graduating with a “very good (> 2.99)” degree most is gender. This result is in line with many studies that advocate that being a woman causes a positive and significant effect on performance [38–40]. Women’s participation in higher education has been increasing for years in Turkey. Some studies [28] in the literature determined that the increase in women’s participation in higher education narrowed down the difference in performance-based bias on gender and this increase shows that there is no decrease in quality and ability.

The results revealed that there was a marginal increase in grades in the medical training in Turkey even after the other factors that might affect the graduation grades were taken under control. A 31% grade inflation (from 2.41 to 3.16) within the past 15 years is the highest value that is reported in the literature. A series of studies documented the increase in average undergraduate grades in the last half-century. Long term analyses from 1960 to 2006 on the grade inflation indicated that the average grades rose from 2.5 to 3.1 (4-point rating scale) at US universities where the grade inflation studies are the most common [37, 41]. Again, Rojstaczer and Healy [15, 41] stated an increase of roughly 0.1 (0 to 4) in every decade since the 1960s. Specifically, it is found that from 1960, the grades increased roughly 0.7 on average in non-profit private universities and 0.5 in public universities. Similarly, Summary and Weber [42] found that the average grade of 2.6 (GPA) at a university in southeastern Missouri increased to 3.1 in 2004. Similarly, the grades rose from 2.83 to 2.97 between 1993 and 2004 at another university [43]. Carter and Lara [14] reported a tendency to increase grade distributions in the US at the University of California (UC) and the California State University (CSU) campuses between 2009 and 2013, reporting a significant increase in GPA in only half of the UC campuses during this time. Although the size of the grade inflation varies according to the data sources, evidence shows that grades increase around 0.1 every decade. Calculating the grade-inflation based on only grade calculations may cause errors. Some potential factors in the context of years lead to an increase in grade. Increased student diversity, new curriculum or grading policies, importance of student assessments, improvement in quality of education; and improvement in teaching skills of instructors can be examples for these factors [13, 22, 23].

Some of the researchers do not believe that there is grade inflation. Mostrom and Blumberg [44] stated that the rise of grades does not necessarily mean inflation, and they argue that what could be happening because of performance improvement (that is what they assume). The instructors who describe the requirements of the course, the evaluation lists, and the interest of students in the classroom are more likely to get students to learn and therefore receive higher grades. Again, Summary and Weber [42] state that grade change is not due to inflation, but due to productivity improvements, which naturally increases students’ learning and understanding. But the analyses showed that after these factors were controlled, the average increase in grades was roughly half the unconditional increase [37].

The highest-grade inflation in the pre-graduation medical training in Turkey yearly was detected between 2017 and 2020. Additionally, there is a quite high-grade inflation increase in 2017. There was a coup attempt on 15 July 2016 in Turkey. Fifteen non-profit private universities were closed on 23 July 2016 under the cover of the Gulenist (FETO) terrorist group who attempted the coup, and the students were transferred to other universities. The result obtained in 2017 can be explained by the fact that these medical students, who completed the majority of their studies in the previously closed non-profit private universities, completed their senior year in public universities (according to analyses, the graduation grades are quite higher in non-profit private universities than public universities), and this reflected on their grades.

Unlike previous studies [28, 45], a negative correlation was found between the university entrance scores and the percentage of graduating with a “very good” degree. There are many possible explanations for this. University placement processes are carried out centrally in Turkey. As a reflection of this situation, candidate students tend to make university selections based on previous rankings. One of the most important indicators that determine this ranking is the positive perception towards the universities and medical schools in society. This situation may create pressure over school administrations and instructors, which have a tradition of admitting students with very high scores, and this implicitly causes lower grade policies. Therefore, this result obtained in this study is an expected result for Turkey although it is not in compliance with the international literature.

The possibility of graduating with a “very good” degree is quite high both in non-profit private universities and in young public medical schools. These results are in parallel with the findings in the literature. Unlike public universities which are funded fully by the government, university administrations may use grades as a tool to keep students in non-profit private universities, which are financed by the payments of students [4]. In fact, schools tend to make students and parents happy as they want to get high grades. One method to maintain students’ happiness is to give them good grades [46]. Similarly, some studies revealed that the reputations of the universities that graduate their students with high degrees are not better than universities that graduate their students with low degrees [47]. Especially established universities are aware of grade inflation and take measures against this. For example, Princeton University declared that they will not allow more than 35% of the grades to be A in all departments [48].

Furthermore, the results of the study confirm some other key findings in the literature. To illustrate, the academic background of the instructor, the class level, academic fields, and the university entrance scores are observed as substantial determinants of the average course grades. In the previous parts of this study, it was mentioned that the grades were marginally inflated, and the grade inflation was quite high. Several factors of this marginal increase were determined in the analyses. To exemplify, the fewer students in the class, the higher the average grade was, and this finding is in line with the findings in the literature [32, 34]. It proves that if the class size is small, the instructors have opportunities to know students better and to give more feedback to their homework, etc. Also, the more instructors know about the student’s effort, the more flexible they tend to be. Even, just having a closer relationship could make the instructors less likely to give a “bad” grade because of the fear of disappointing the learners [49].

In conclusion, it is very clear that the changes in grades observed in this study is a result of “grade inflation” rather than the increasing efforts and works of students or more qualified student selection. Because in 2005 which is the starting point of the study total quota of medical schools in Turkey was around five thousand, while in 2020 it increased up to 16 thousand within years. This situation is an indicator of that points of placement (of quality) of medical school students in Turkey where a central entrance exam is administered have decreased compared to previous years. Therefore, when both the decline in placement performance of students in time and statistical control variables are considered in the study, the cause of grade inflation in medical education in Turkey does not stem from better performance of the medical students. However, this study only reflects the pre-graduation medical training in Turkey. Additionally, the period determined for this analysis covers 15 years, and a limited number of factors that are considered to affect grades were checked. These factors do not include a detailed description of the learning, teaching and assessment strategies used in distance education courses, as well as a long period of identification of individual characteristics of the educators. The inclusion of these factors in empirical analysis will be productive for future research. In the future, it will also be interesting to study above variables in the context of various countries’ medical schools.

## Data Availability

The datasets used and analyzed during the current study are available from the corresponding author on reasonable request.
